# Induction Chemotherapy and Ablative Stereotactic Magnetic Resonance Image-Guided Adaptive Radiation Therapy for Inoperable Pancreas Cancer

**DOI:** 10.3389/fonc.2022.888462

**Published:** 2022-06-23

**Authors:** Michael D. Chuong, Roberto Herrera, Adeel Kaiser, Muni Rubens, Tino Romaguera, Diane Alvarez, Rupesh Kotecha, Matthew D. Hall, James McCulloch, Antonio Ucar, Fernando DeZarraga, Santiago Aparo, Sarah Joseph, Horacio Asbun, Ramon Jimenez, Govindarajan Narayanan, Alonso N. Gutierrez, Kathryn E. Mittauer

**Affiliations:** ^1^ Department of Radiation Oncology, Miami Cancer Institute, Miami, FL, United States; ^2^ Herbert Wertheim College of Medicine, Florida International University, Miami, FL, United States; ^3^ Office of Clinical Research, Miami Cancer Institute, Miami, FL, United States; ^4^ Department of Medical Oncology, Miami Cancer Institute, Miami, FL, United States; ^5^ Department of Surgical Oncology, Miami Cancer Institute, Miami, FL, United States; ^6^ Department of Interventional Oncology, Miami Cancer Institute, Miami, FL, United States

**Keywords:** pancreas cancer, ablative, radiotherapy, magnetic resonance image, chemotherapy

## Abstract

**Background:**

Radiation therapy (RT) dose for inoperable pancreatic ductal adenocarcinoma (PDAC) has historically been non-ablative to avoid injuring gastrointestinal (GI) organs at risk (OARs). Accruing data suggest that dose escalation, in select patients, may significantly improve clinical outcomes. Early results of ablative stereotactic magnetic resonance image-guided adaptive radiation therapy (A-SMART) have been encouraging, although long-term outcomes are not well understood.

**Methods:**

A single institution retrospective analysis was performed of inoperable non-metastatic PDAC patients who received induction chemotherapy then 5-fraction A-SMART on a 0.35T-MR Linac from 2018-2021.

**Results:**

Sixty-two patients were evaluated with a median age of 66 years (range 35-91) and nearly all achieved Eastern Cooperative Oncology Group (ECOG) performance status 0-1 (96.8%). Locally advanced disease was common (72.6%), otherwise borderline resectable (22.6%), or medically inoperable (4.8%). All received induction chemotherapy for a median 4.2 months (range, 0.2-13.3) most commonly FOLFIRINOX (n=43; 69.4%). Median prescribed dose was 50 Gy (range 40-50); median biologically effective dose (BED_10_) was 100 Gy_10_. The median local control (LC), progression-free survival (PFS), and overall survival (OS) from diagnosis were not reached, 20 months, and 23 months, respectively. Also, 2-year LC, PFS, and OS were 68.8%, 40.0%, and 45.5%, respectively. Acute and late grade 3+ toxicity rates were 4.8% and 4.8%, respectively.

**Conclusions:**

To our knowledge, this is the largest series of induction chemotherapy followed by ablative 5-fraction SMART delivered on an MR Linac for inoperable PDAC. The potential for this novel treatment strategy is to achieve long-term LC and OS, compared to chemotherapy alone, and warrants prospective evaluation.

## Introduction

The prognosis of patients with inoperable pancreatic ductal adenocarcinoma (PDAC) is dismal despite substantial efforts to meaningfully improve outcomes (1). Over the last decade, modest gains have been realized by intensifying chemotherapy although long-term local control (LC) and overall survival (OS) are rarely achieved (2–4). Conversely, significantly escalating radiation therapy (RT) to an ablative dose has not been considered feasible for most patients using conventional image guidance because of interfraction anatomic changes and uncertainty in assuring that dose to nearby organs at risk (OARs) is safe prior to delivering each fraction (5).

In recent years, the hypothesis that ablative radiation dose may improve long-term OS has garnered increasing attention (6–11). Stereotactic magnetic resonance image-guided adaptive radiation therapy (SMART) is particularly well suited for dose escalation, especially to mobile tumors in the abdomen and pelvis, because of its unique imaging and online adaptive replanning capabilities (9–13). A multi-institutional retrospective analysis by Rudra and colleagues demonstrated that dose escalation above a biologically effective dose (BED_10_) >70 Gy_10_ using a 0.35 Tesla (T) magnetic resonance (MR)-guided cobalt-60 treatment machine was associated with significantly improved OS (9). Subsequent single institution experiences of ablative SMART (A-SMART) prescribed up to 50 Gy in 5 fractions (BED_10_ = 100 Gy_10_) have also demonstrated minimal grade 3 or higher toxicity and favorable early efficacy (10, 14).

While these data are encouraging, there is a paucity of published outcomes of ablative RT for inoperable PDAC with extended follow-up. Therefore, we performed an updated analysis of our previously published institutional experience of A-SMART for inoperable PDAC (11).

## Materials and Methods

### Patient Selection and Staging

After obtaining institutional review board (IRB) approval, we performed a single institution retrospective analysis of patients treated on the MRIdian Linac (ViewRay, Oakwood Village, OH) between 2018-2021 for non-metastatic PDAC.

Patients were staged with endoscopic ultrasound and computerized tomography (CT) scans. Most also had MR scans of the abdomen although positron emission tomography (PET) scans were not routinely used for initial staging. Resectability was determined according to the National Comprehensive Cancer Network (NCCN) criteria (15).

Only patients who received induction chemotherapy were included. A-SMART was considered if restaging studies showed no evidence of distant progression. There was not a maximum tumor size or minimum distance between gross tumor and gastrointestinal (GI) organs at risk (OARs) for patients to be offered A-SMART. As such, even patients with extensive abutment of gross disease and GI OARs were treated with A-SMART. Conversely, A-SMART was not offered if there was duodenal invasion by tumor as seen on endoscopic evaluation. No patient had prior abdominal RT. Patients were not routinely prescribed prophylactic proton pump inhibitors.

### Radiation Therapy Planning and Delivery

Our treatment planning and delivery approach has been previously published (11). Simulation and treatment were done in the supine position typically with both arms down at the sides for comfort and reproducibility. Fiducial markers and intravenous/oral contrast were not used because gross disease and surrounding OARs could be distinctly visualized during treatment using continuous cine-MR imaging. Simulation included a 0.35 T mid-inspiration breath hold and balanced steady-state free precession sequence (TrueFISP) MR scan acquired over 17-25 seconds on the MRIdian Linac. This was followed by a simulation CT scan.

**Figure 1:**
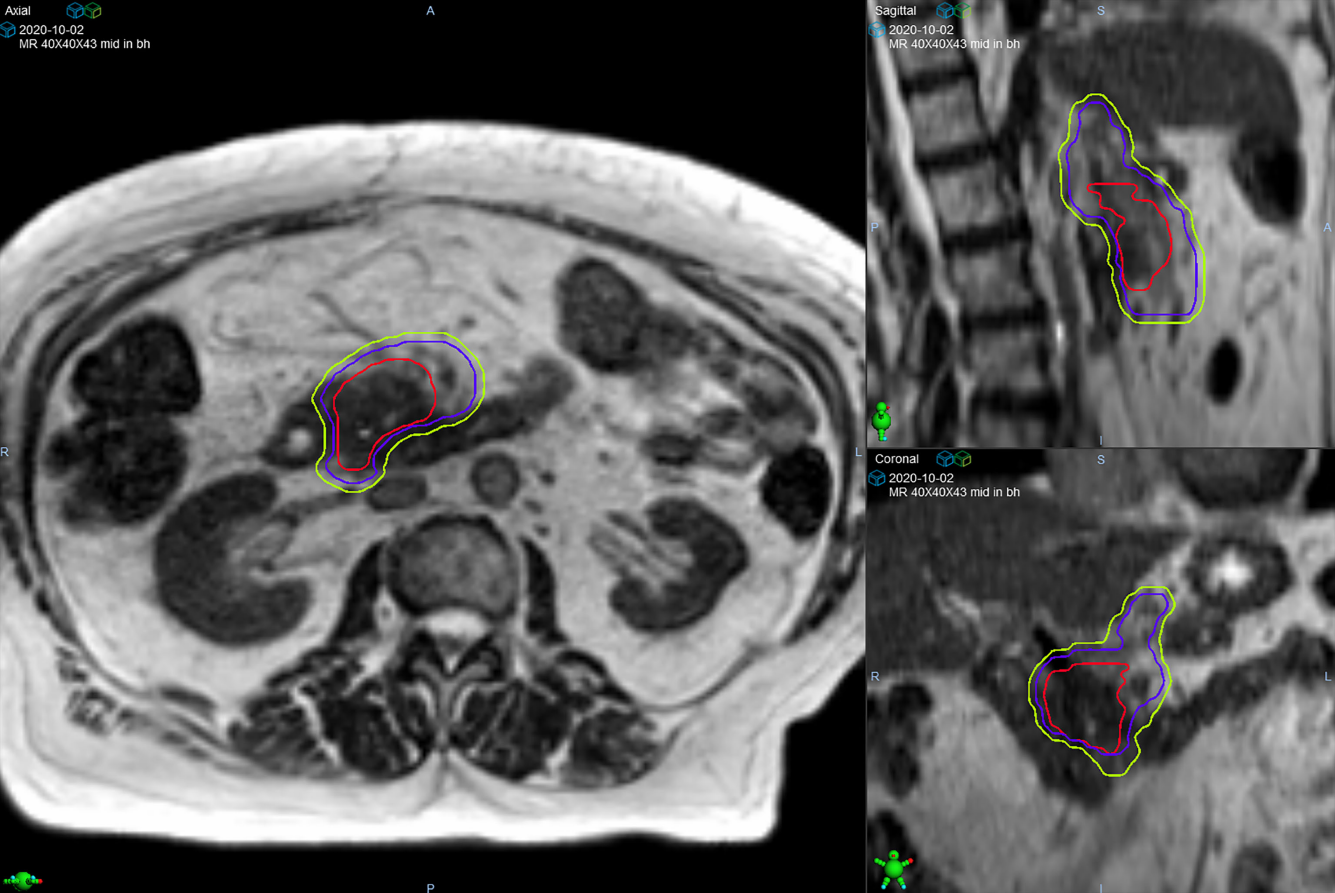
Target volumes of a patient with pancreatic head/uncinate process lesion who was prescribed 50 Gy in 5 fractions. The gross tumor volume (red line) is surrounded by the clinical target volume (purple line) that includes the celiac axis and superior mesenteric artery. The planning target volume (green line) was created from a 3 mm expansion of the clinical target volume.

Target delineation and OAR segmentation were defined on the MR simulation scan, which was the primary scan for treatment planning. Contouring of GI OARs was done ensuring that the full thickness of the muscular wall in addition to the lumen of each structure was included. The gross tumor volume (GTV) included all visible tumor within the pancreas and any involved regional lymph nodes. After we gained confidence that ablative dose delivered to gross tumor alone was tolerated well, in late 2019 there was a systematic shift to routinely include a clinical target volume (CTV) that included a 5 mm isotropic margin around the GTV, proximal ~3 cm of the celiac axis (CA) and superior mesenteric artery (SMA) (**Figure 1**). Based on physician preference, the elective region was prescribed the same dose as the GTV (n=36; 61%) or a lower dose (33-35 Gy) in 5 fractions using a simultaneous integrated boost (SIB) (n=23; 39%). The planning target volume (PTV) was created through an isotropic 3 mm expansion of the GTV, or CTV if present, for all patients. The PTVs for the SIB approach were denoted as PTV50 and PTV33-35 to differentiate the ablative and lower dose levels. A 120-140% hotspot was intentionally delivered to as much of the GTV as possible. The highest priority for all delivered plans was to ensure that OAR constraints were met (stomach, duodenum, small bowel: V35 ≤0.5 cc, V40 ≤0.03 cc; large bowel: V38 ≤0.5 cc, V43 ≤0.03 cc; liver mean ≤15 Gy; kidneys mean ≤10 Gy; spinal cord V25 V25 ≤0.03 cc), even if this meant sacrificing target coverage (11). We used an isotoxicity planning approach to maximize target coverage, by which treatment plans were normalized to the nearest GI OAR.

All patients were treated with continuous cine-MR imaging and real-time tissue tracking with automatic beam gating. Prior to each daily treatment, the GTV was used to define the tracking region of interest (known as the “tracking structure”) in the sagittal plane and treatment was automatically held when >3-5% was displaced >3 mm from its original position (i.e., outside of the “tracking boundary”). Mid-inspiration breath hold was preferred over free breathing respiratory gating to improve the duty cycle efficiency and decrease the time that the patient was required to be in the treatment machine. On-table adaptive replanning was performed if deemed medically necessary based on the predicted dose (i.e., the dose resulting from the initial plan recalculated on the anatomy of the day). The highest priority during both initial planning and adaptive re-planning was to ensure all OAR constraints were met, and then secondarily optimization of target volume coverage by the prescription dose.

### Post-SMART Evaluation and Additional Therapy

Follow-up consisted of physical examination, CT scans (chest, abdomen, pelvis), and labs including CA19-9 at 4-6 weeks after SMART and otherwise at approximately 3-month intervals. We did not evaluate patients prior to 4 weeks because CA19-9 could potentially be transiently elevated from treatment rather than disease progression. PET/CT scans were not routinely ordered although were occasionally acquired to further investigate findings from CT and/or magnetic resonance image (MRI) scans. Treatment response was assessed using Response Evaluation Criteria in Solid Tumors (RECIST) version 1.1 criteria. Toxicity outcomes were prospectively recorded at least once during SMART and then at each follow up visit using Common Terminology Criteria for Adverse Events (CTCAE) version 5.

Chemotherapy after SMART was given at the discretion of the treating medical oncologist, although in general was not recommended unless there was concern for or definitive evidence of tumor progression based on radiographic findings and/or CA19-9 change. Patients were offered surgery based on multi-disciplinary tumor board discussion after SMART and this was intended to be done within 8 weeks after SMART, if possible.

### Outcomes Assessment

LC was defined as absence of in-field treatment failure. Progression free survival (PFS) was defined as the time to local progression, distant progression, or death. OS was determined to be the time to death or otherwise last follow-up.

Common Terminology Criteria for Adverse Events (CTCAE version 5.0) was used to evaluate toxicity. Acute toxicity was considered to have occurred during or within 90 days from the beginning of SMART. Toxicity was prospectively recorded in the electronic medical record at the time of each clinic encounter.

### Statistical Evaluation

The Research Electronic Data Capture system was used to collect and manage data. Median and range for continuous variables and frequencies and percentages for categorical variables were used for describing patient, tumor, and treatment characteristics. Clinical outcomes were evaluated using the Kaplan-Meier method. Treatment response was determined according to the Response Evaluation Criteria in Solid Tumors 1.1. Patients were censored at the date of last follow-up who were alive and did not experience tumor progression. The Kaplan-Meier method was used to determine estimated LC, PFS, and OS. A Cox proportional hazards model was used to evaluate prognostic factors of LC, PFS, and OS in univariate (UVA) and multivariate analyses (MVA). All variables with *P*<0.10 in the univariate analysis were entered in the multivariate model. Statistical significance was set at *P*<0.05. Statistical analysis was performed using SAS (version 9.4, SAS Institute, Cary, NC).

## Results

### Patient, Tumor, and Treatment Characteristics

Sixty-two patients were evaluated (**Table 1**), most with tumors in the head of pancreas (n=55; 88.7%). Nearly all had Eastern Cooperative Oncology Group (ECOG) performance status 0-1 (n=60; 96.8%). The median largest tumor dimension after induction chemotherapy and prior to A-SMART was 3.8 cm (range, 1.5-6.9 cm). The majority had locally advanced disease (n=45; 72.6%) while borderline resectable (n=14; 22.6%) and resectable but medically inoperable (n=3; 4.8%) PDAC were less common.

**Table 1:** Patient, tumor, and treatment characteristics.

Characteristic	N (range)
Total number of patients	62
Age (year), median	66 (35-91)
Gender Male Female	35 (59.3%) 24 (40.7%)
ECOG performance status 0-1 2	60 (96.8%) 2 (3.2%)
Histology Adenocarcinoma	62 (100%)
Tumor location Head Body/tail	55 (88.7%) 7 (11.3%)
Largest tumor size (cm), median	3.8 (1.5-6.9)
Resectability Locally advanced Borderline resectable Resectable, medically inoperable	45 (72.6%) 14 (22.6%) 3 (4.8%)
Clinical T stage 1 2 3 4	1 (1.6%) 13 (21.0%) 9 (14.5%) 39 (62.9%)
Clinical N stage 0 1 2	43 (69.4%) 18 (29.0%) 1 (1.6%)
Clinical M stage 0	62 (100%)
CA 19-9 (U/mL), median Initial diagnosis Before SMART	168.7 (0.9-12,868.6) 45.2 (1-3686)
Induction chemotherapy regimen FOLFIRINOX Gemcitabine/nab-paclitaxel Gemcitabine	43 (69.4%) 15 (24.2%) 4 (6.4%)
Induction chemotherapy duration (months), median	4.2 (0.2-13.3)
Radiation dose Total prescribed dose (Gy), median Total prescribed fractions	50 (40-50) 5
Elective volume coverage Yes No	50 (80.6%) 12 (19.4%)
Post-SMART therapy Surgery Irreversible electroporation Chemotherapy	14 (22.6%) 6 (9.7%) 32 (51.6%)

ECOG, Eastern Cooperative Oncology Group; SMART, stereotactic magnetic resonance-guided adaptive radiation therapy; GTV, gross tumor volume.

Induction chemotherapy was given to all patients, most commonly FOLFIRINOX (n=43; 69.4%) or gemcitabine/nab-paclitaxel (n=15; 24.2%), for a median 4.2 months (range, 1.2-13.3 months). The median CA19-9 at diagnosis was 168.7 U/mL (range, 0.9-12,868.6 U/mL) that decreased after chemotherapy to a median 45.2 U/mL (range, 1-3686 U/mL).

The median prescribed radiation dose was 50 Gy (range, 40-50 Gy) delivered in 5 consecutive fractions. In our early experience a few patients were prescribed 40 Gy (n=2; 3.2%) or 45 Gy (n=5; 8.1%), and when we did not observe severe toxicity from these doses, we increased to 50 Gy (n=55; 88.7%) that since has been routine. The prescription dose was delivered to most of the target volumes on the initial plan created from the simulation day anatomy despite the proximity of GI OARs, and this coverage was similar across the adapted fractions while ensuring that all GI OAR constraints were met (**Table 2**). The median GTV D_90_ on the original versus adapted plans was 48.1 Gy and 48.4 Gy, respectively. The median PTV50 D_90_ on the original versus adapted plans was 47.2 Gy and 46.2 Gy, respectively.

**Table 2:** Target volume coverage on the initial plan versus the on-table adaptive plans.

Target Volume	Initial plan dose (Gy)from simulation anatomy			On-table adaptive plan dose (Gy)from treatment day anatomy		
	Median	Mean ± SD	Range	Median	Mean ± SD	Range
GTV D_90_	48.1	48.9 ± 5.3	36.6-60.5	48.4	48.6 ± 5.2	36.5-61.0
GTV D_80_	52.0	52.0 ± 4.8	41.2-61.6	51.4	51.4 ± 4.6	40.6-61.5
CTV D_90_	42.8	44.5 ± 6.7	30.1-56.0	44.9	44.2 ± 5.9	31.3-55.0
CTV D_80_	49.9	48.8 ± 6.5	33.9-59.0	50.5	48.4 ± 5.5	33.8-56.9
PTV33-35 D_90_	39.2	40.7 ± 6.6	24.0-53.0	39.3	39.7 ± 6.1	25.1-60.8
PTV33-35 D_80_	44.7	45.2 ± 6.1	28.2-54.9	45.0	44.2 ± 5.6	29.7-63.3
PTV50 D_90_	47.2	46.9 ± 5.0	33.2-55.4	46.2	45.8 ± 5.5	33.2-94.4
PTV50 D_80_	50.0	49.4 ± 4.6	37.-56.5	48.7	48.2 ± 4.2	37.0-63.3

D_90_, dose to 90% of the volume; D_80_, dose to 80% of the volume; GTV, gross tumor volume; CTV, clinical target volume; PTV, planning target volume.

**Figure 2:**
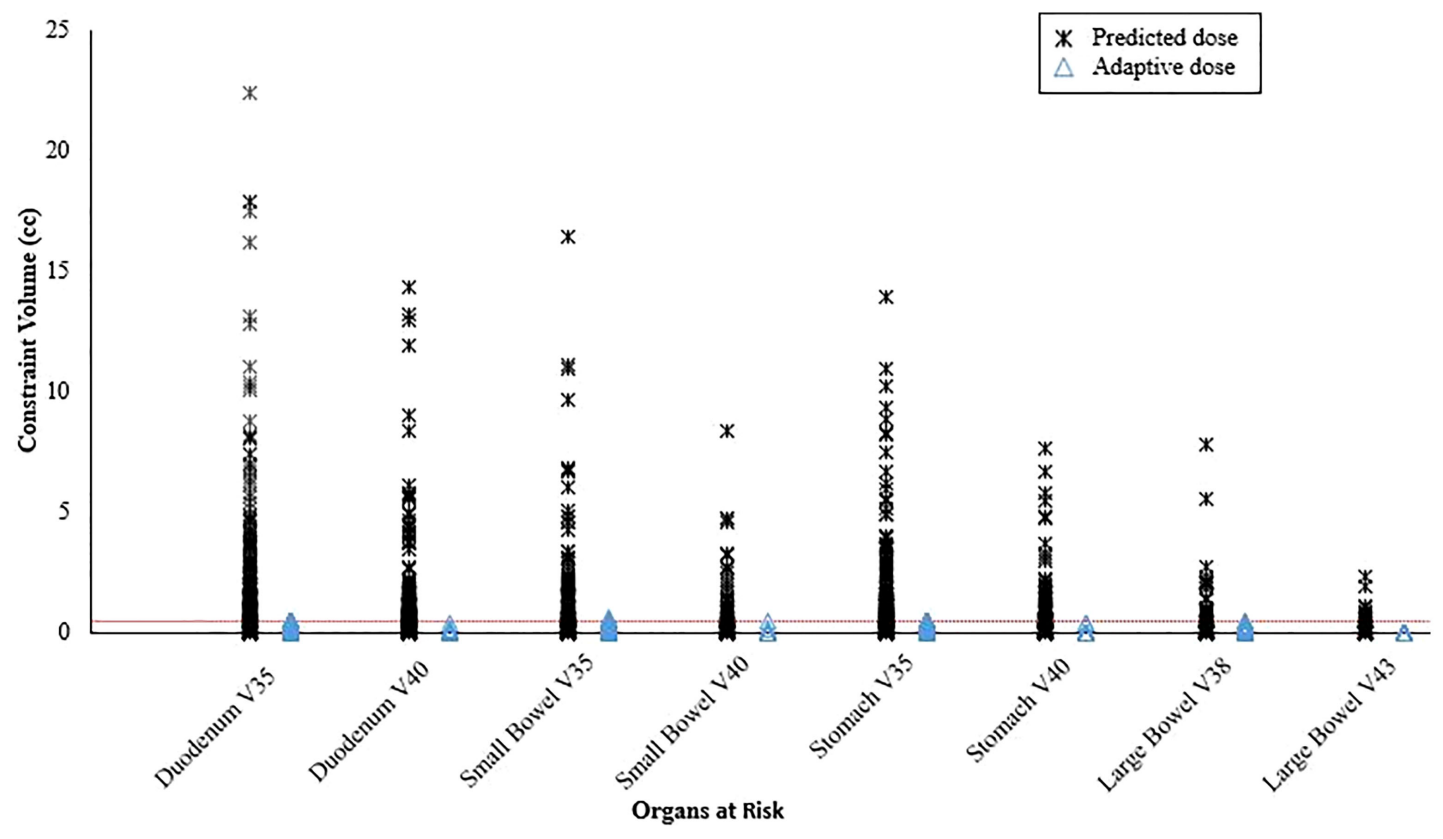
Predicted organ at risk dose/volumes assuming the original plan was used on the day of treatment anatomy compared to on-table adaptive replanning that was able to ensure all dose constraints were met for every fraction. The horizontal dotted line represents the constraint volume of 0.5 cc.

Online adaptive replanning was performed for 5 fractions in nearly all patients (n=58; 93.5%) and was indicated because of predicted GI OAR constraint violations (**Figure 2**). Only 2 of our first patients were treated without adapted fractions; and both were prescribed 40 Gy to gross disease only.

### Additional Therapy After A-SMART

Surgery was performed in 14 patients (22.6%) after a median 10.7 weeks (range, 5.6-44.1 weeks) from A-SMART, 10 (71.4%) with borderline resectable, and 4 (28.6%) locally advanced PDAC at initial diagnosis. Resection and reconstruction of the superior mesenteric vein/portal vein was done in 7 (50%) patients; none had resection of the CA or SMA. All received FOLFIRINOX (n=13; 92.9%) or gemcitabine/nab-paclitaxel (n=1; 1.6%), for a median 4.7 months (range, 1-8.1 months). The prescribed radiation dose was 40 Gy (n=1; 7.1%) or 50 Gy (n=13; 92.9%). Nearly all (n=12; 85.7%) had radiographic stable disease after A-SMART, yet all had significant histopathologic response in the primary lesion (1 ypT0, 11 ypT1, 2 ypT2) and 13 (92.9%) had negative lymph nodes. Thirteen (92.9%) achieved negative surgical margins.

Irreversible electroporation (IRE) was performed in 6 patients (9.7%) at a median 9.6 months (range, 2.3-29.0 months) after A-SMART. The most common indication was regional disease recurrence outside of the treatment field without distant progression (n=5); one patient did not have increasing CA19-9 or radiographic evidence of progressive disease although had stable disease by RECIST that was considered, by tumor board consensus to possibly represent an incomplete response to A-SMART.

Chemotherapy was typically not resumed after A-SMART unless there was radiographic evidence of disease progression and/or increasing CA19-9. As of the last follow-up date, 32 (51.6%) patients had not resumed chemotherapy.

### Disease Control and Survival

Median follow-up for all patients was 18.6 months (interquartile range [IQR], 6.8-44.9 months) from diagnosis and 11.0 months (IQR, 1.5-36.0) from start of A-SMART. At the time of analysis, 23 patients (37.1%) were still alive.

**Figure 3:**
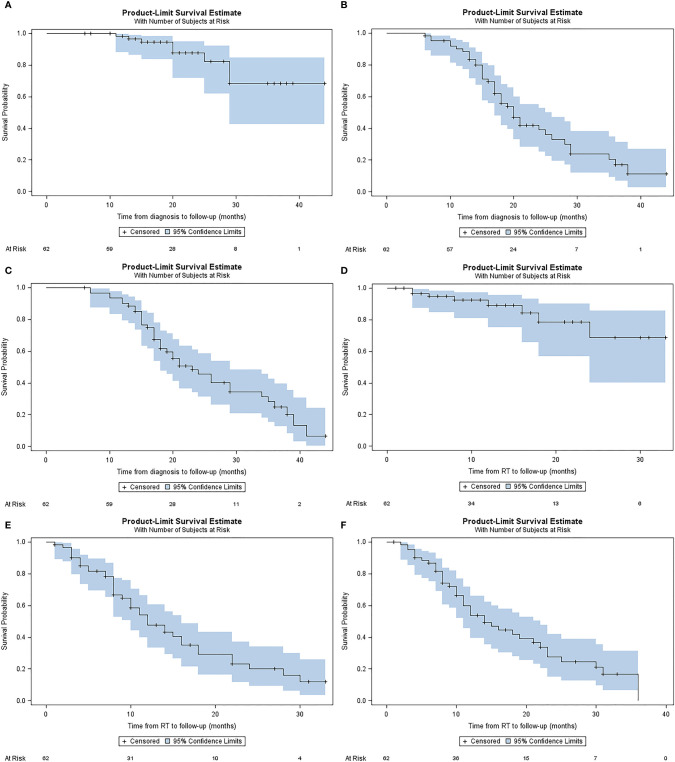
Kaplan-Meier plots describing estimated **(A)** local control from diagnosis, **(B)** progression free survival from diagnosis **(C)** overall survival from diagnosis, **(D)** local control from A-SMART, **(E)** progression free survival from A-SMART, **(F)** overall survival from A-SMART.

Median LC from diagnosis was not reached. 1- and 2-year LC from diagnosis were 98.3% (IQR, 94.8-100%), and 87.7% (IQR, 77.0-98.3%), respectively (**Figure 3A**). Median PFS from diagnosis was 20 months (IQR, 17.0-25.0). 1- and 2-year PFS from diagnosis were 88.4% (IQR, 80.4-96.5%), and 40% (IQR, 25.8-54.2%), respectively (**Figure 3B**). Median OS from diagnosis was 23 months (IQR, 18.0-29.0). 1-year, and 2-year OS from diagnosis were 90.2% (IQR, 82.7-97.6%), and 45.5% (IQR, 31.5-59.5%), respectively (**Figure 3C**).

Median LC after A-SMART was not reached. 1- and 2-year LC after A-SMART were 98.2% (IQR, 79.8%-98.6%), and 68.8% (IQR, 45.9%-91.7%), respectively (**Figure 3D**). Median PFS after A-SMART was 12 months (IQR, 10.0-16.0). 1- and 2-year PFS after A-SMART were 49.0% (IQR, 35.1-62.95%), and 20.6% (IQR, 7.5-33.7%), respectively (**Figure 3E**). Median OS from A-SMART was 14 months (IQR, 11.0-22.0). 1-year, and 2-year OS from A-SMART were 53.8% (IQR, 40.3-67.4%), and 27.7% (IQR, 13.9-41.5%), respectively (**Figure 3F**).

The percentage CA19-9 change after induction chemotherapy and prior to A-SMART was the only significant prognostic factor for OS on multivariate analysis (hazard ratio 1.005; 95% confidence interval 1.001-1.009; P=0.008) (**Table 3**).

**Table 3:** Multivariate analyses of factors predicting for overall survival.

Variables	Univariate		Multivariate	
	HR (95% CI)	*P* value	HR (95% CI)	*P* value
Age	1.009 (0.982, 1.036)	0.510		
Sex (female versus male [ref])	0.501 (0.214, 1.172)	0.110	0.546 (0.223, 1.337)	0.185
Location (body versus head [ref])	1.043 (0.313, 3.482)	0.944		
ECOG (1-2 versus 0 [ref])	1.911 (0.875, 4.174)	0.104	2.168 (0.943, 4.986)	0.068
T stage (1-3 versus 4 [ref])	1.446 (0.583, 3.586)	0.425		
N stage (1-2 versus 0 [ref])	1.214 (0.554, 2.659)	0.627		
Induction chemo drug (other versus Folfirinox [ref])	1.542 (0.754, 3.15)	0.235		
Induction chemo duration (>median versus <median [ref])	0.99 (0.492, 1.988)	0.976		
CA 19-9% change	1.004 (1.001, 1.008)	0.011	1.005 (1.001, 1.009)	0.008
Change in CA 19-9 (increase versus decrease [ref])	0.938 (0.452, 1.947)	0.864		
GTV volume (>median versus <median [ref])	2.407 (1.112, 5.21)	0.025	0.877 (0.387, 1.99)	0.753
GTV dose (>median versus <median [ref])	1.109 (0.539, 2.281)	0.779		
PTV volume (>median versus <median [ref])	2.335 (1.109, 4.916)	0.025	1.457 (0.628, 3.376)	0.380
PTV dose (>median versus <median [ref])	0.717 (0.331, 1.553)	0.398		
Elective coverage (yes versus no [ref])	0.649 (0.226, 1.863)	0.422		
Surgery (yes versus no [ref])	0.841 (0.413, 1.713)	0.633		
Post-RT chemo (yes versus no [ref])	1.009 (0.982, 1.036)	0.510		

HR, hazard ratio, CI, confidence interval; RT, radiation therapy; ECOG, Eastern Cooperative Oncology Group; GTV, gross tumor volume; PTV, planning target volume; A-SMART, ablative stereotactic magnetic resonance image guided radiation therapy.

There was no statistically significant difference in LC from diagnosis based on surgery versus no surgery (not reached for both). Median PFS from diagnosis was shorter in patients who did not have surgery (18 vs. 35 months; P=0.06) due to more rapid distant progression; patients who had surgery had numerically higher median OS although the difference was not statistically significant (median 35 vs. 21 months; P=0.27).

### Treatment-Related Toxicity

The delivery of ablative dose did not cause significant toxicity in most patients. Acute grade 3 toxicity (4.8%) included duodenal stenosis requiring stenting in 2 patients with tumor in the head of pancreas abutting the second/third part of the duodenum and one patient with abdominal pain lasting several hours after receiving the first fraction that resolved with medication and did not recur. There was no acute grade 4-5 toxicity. Late grade 3+ toxicity (4.8%) consisted of 2 patients with grade 3 GI bleed that resolved with transfusion. One patient’s status post Whipple procedure 7 weeks after A-SMART, with an initially unremarkable postoperative course, died 6 weeks later due to a gastroduodenal artery bleed not definitely related to A-SMART (possible grade 5).

## Discussion

To our knowledge, this is the largest published experience of A-SMART delivered in 5 consecutive fractions for inoperable PDAC. Building on our initial clinical experience of 35 patients (11), the current analysis included 62 patients who all received induction chemotherapy and achieved median and 2-year OS from diagnosis of 23 months and 45.5%, respectively. These outcomes add to a small, yet growing, body of literature suggesting that radiation dose escalation could be associated with improved OS for patients with inoperable PDAC (7–11). Most recently, Reyngold and colleagues evaluated 119 locally advanced PDAC patients who received induction chemotherapy and ablative RT in 15 (19%) or 25 (81%) fractions delivered using CT guidance and median OS from diagnosis and RT were 26.8 and 18.4 months, respectively (8). While no study has prospectively compared outcomes based on prescribed dose for inoperable PDAC, LC, and OS after ablative RT are seemingly higher than what is has been reported after non-ablative dose (7, 16, 17). We must acknowledge the potential effect of evolving chemotherapy regimens on improving clinical outcomes including OS and, therefore, prospective evaluation is needed to better understand the impact of radiation dose escalation when delivered after contemporary multi-agent chemotherapy. However, outcomes from the recently published LAPC-1 trial that included FOLFIRINOX for 8 cycles then 40 Gy in 5 fractions suggest that there is a potential role for radiation dose escalation; 2-year LC from chemotherapy was ~60% (versus 87.7% in our study) and median OS was 15 months in unresected patients (versus 21 months in our study) (16).

Why might radiation dose escalation impact OS? About one-third of PDAC-related deaths are due to local progression (18), and it is by delaying or preventing these deaths through radiation dose intensification that long-term OS might be improved, at least for select patients. While a radiation dose response relationship with LC has been demonstrated (19), the modest improvement in LC achieved when using RT versus chemotherapy alone has not translated into improved OS as demonstrated in the LAP07 trial likely because non-ablative dose is not sufficient to achieve durable LC (20). Conversely, ablative radiation dose achieves excellent long-term LC as demonstrated in the current analysis where the median LC was not reached and 2-year LC from the start of A-SMART approached 70% despite some tumors measuring up to almost 7 cm. Similar outcomes have been reported in other recently published dose-escalated RT studies (8, 9). Of note, elective volume/nodal irradiation has increasingly been adopted as treatment for pancreas SBRT, including our institution; and recent data published by the Stanford group suggests that this at least improves PFS, although further evaluation is needed (21–23).

The emergence of MR guidance has led to a fundamental shift in how RT is delivered for some cancers (24). SMART provides superior soft tissue image quality, real-time continuous intrafraction cine-MRI, soft tissue tracking, and automatic beam gating, which are critical to ensuring that OAR constraints are met while delivering ablative dose, especially with ultra-hypofractionation, to most, if not all, of the target (11). In addition, an MR Linac enables rapid online adaptive replanning that allows for OAR constraints to be met and target volume coverage to be maximized despite interfraction anatomic changes by reoptimizing the original plan to account for the current day’s anatomy (25). In the current analysis, we demonstrated that treating with the original plan would have violated at least one GI OAR constraint for nearly all fractions and that treating with an adaptive plan resulted in all constraints being met (**Figure 3**). SMART also seems to achieve safe dose escalation in only 5 fractions whereas a more fractionated course (e.g., 15-25 fractions) is likely needed if using CT guidance without adaptive replanning (8). A limitation of the adaptive workflow is that it requires additional time and resources, although we believe this can be justified by the seemingly large gains in treatment efficacy.

Topics of interest that deserve the attention of future studies include the development of novel prognostic biomarkers to better identify patients who should receive local therapy in addition to systemic therapy (26). It is not uncommon for some patients to experience rapid distant progression even after receiving extended chemotherapy and these patients presumably would be less likely to achieve meaningful long-term benefit from ablative RT. Assessing response after A-SMART is also currently challenging since “stable disease” can be misinterpreted on CT and MRI scans as lack of favorable response. Nearly all patients in the current analysis, who had surgery, achieved a significant histopathologic response, yet nearly all did not have any significant radiographic change, demonstrating that radiographic response is not adequate in itself to assess local treatment effect. Moreover, the discrepancy between radiographic and pathologic outcomes after preoperative therapy are well documented (27). Lastly, the cumulative dose delivered across all adapted fractions is not readily assessable on any commercially available MR Linac. Cumulative delivered dose may be associated with treatment efficacy and safety and may be useful to consider when optimizing each adapted fraction to improve the therapeutic ratio (14).

There are several limitations of this analysis including its retrospective design, single institution nature, duration of follow-up, and relatively small size. We recognize that retrospective studies may underreport toxicity but attempt to mitigate this by prospectively evaluating toxicity at each patient encounter whenever possible. We did not collect patient-reported outcomes that would have added to our understanding of patient tolerability and effects on quality of life; we plan to assess this in future patients. There was considerable heterogeneity in additional therapy delivered after A-SMART. While we report outcomes in patients who had surgery versus no surgery after A-SMART, there needs to be longer follow up to better understand the potential benefit of surgery. Follow up is also necessary regarding potential risks of operating after the delivery of such a high dose of A-SMART, which include major vascular structure by nature of patients having borderline resectable and locally advanced PDAC (28).

In conclusion, we demonstrate that induction chemotherapy and 5-fraction A-SMART appears to achieve a favorable therapeutic ratio for patients with initially inoperable PDAC, achieving durable LC for most patients and encouraging 2-year OS with minimal severe toxicity. Our findings add to the growing literature in support of significant dose escalation for inoperable PDAC and provide a strong rationale for future prospective evaluation of this novel treatment strategy.

## Data Availability Statement

The raw data supporting the conclusions of this article will be made available by the authors, without undue reservation.

## Author Contributions

The authors confirm contribution to the paper as follows: study conceptualization: MC and KM; data curation: MC and RH; data analysis: MC, AK, MR, and KM; writing – original draft: MC, MR, and KM; manuscript editing and review: all authors. All authors contributed to the article and approved the submitted version.

## Conflict of Interest

RK reports personal fees and non-financial support from Elekta, grants from Novocure, personal fees from Accuray, grants from Blue Earth Diagnostics, grants from Medtronic, grants from AstraZeneca, grants from Exelixis, personal fees from ViewRay, outside the submitted work. AG reports personal fees and non-financial support from ViewRay, outside the submitted work. DA reports grants from Sirtex, outside the submitted work. MC reports grants, personal fees and non-financial support from ViewRay, personal fees from Sirtex, grants from Novocure, personal fees from Advanced Accelerator Applications, outside the submitted work. KM reports personal fees and non-financial support from ViewRay, other from MR Guidance, LLC, outside the submitted work.

The remaining authors declare that the research was conducted in the absence of any commercial or financial relationships that could be construed as a potential conflict of interest.

## Publisher’s Note

All claims expressed in this article are solely those of the authors and do not necessarily represent those of their affiliated organizations, or those of the publisher, the editors and the reviewers. Any product that may be evaluated in this article, or claim that may be made by its manufacturer, is not guaranteed or endorsed by the publisher.

## References

[1] Siegel RL Miller KD Fuchs HE Jemal A. Cancer Statistics, 2021. CA Cancer J Clin (2021) 71(1):7–33. doi: 10.3322/caac.21654 33433946

[2] Walma MS Brada LJ Patuleia SIS Blomjous JG Bollen TL Bosscha K. Treatment Strategies and Clinical Outcomes in Consecutive Patients With Locally Advanced Pancreatic Cancer: A Multicenter Prospective Cohort. Eur J Surg Oncol (2021) 47(3 Pt B):699–707. doi: 10.1016/j.ejso.2020.11.137 33280952

[3] Philip PA Lacy J Portales F Sobrero A Pazo-Cid R Manzano Mozo JL. Nab-Paclitaxel Plus Gemcitabine in Patients With Locally Advanced Pancreatic Cancer (LAPACT): A Multicentre, Open-Label Phase 2 Study. Lancet Gastroenterol Hepatol (2020) 5(3):285–94. doi: 10.1016/S2468-1253(19)30327-9 31953079

[4] Conroy T Desseigne F Ychou M Bouche O Guimbaud R Becouarn Y. FOLFIRINOX Versus Gemcitabine for Metastatic Pancreatic Cancer. N Engl J Med (2011) 364(19):1817–25. doi: 10.1056/NEJMoa1011923 21561347

[5] Courtney PT Paravati AJ Atwood TF Raja N Zimmerman CT Fanta PT. Phase I Trial of Stereotactic Body Radiation Therapy Dose Escalation in Pancreatic Cancer. Int J Radiat Oncol Biol Phys (2021) 110(4):1003–12. doi: 10.1016/j.ijrobp.2021.02.008 33571625

[6] Reyngold M Parikh P Crane CH. Ablative Radiation Therapy for Locally Advanced Pancreatic Cancer: Techniques and Results. Radiat Oncol (2019) 14(1):95. doi: 10.1186/s13014-019-1309-x 31171025 PMC6555709

[7] Krishnan S Chadha AS Suh Y Chen HC Rao A Das P. Focal Radiation Therapy Dose Escalation Improves Overall Survival in Locally Advanced Pancreatic Cancer Patients Receiving Induction Chemotherapy and Consolidative Chemoradiation. Int J Radiat Oncol Biol Phys (2016) 94(4):755–65. doi: 10.1016/j.ijrobp.2015.12.003 PMC4792191 26972648

[8] Reyngold M O'Reilly EM Varghese AM Fiasconaro M Zinovoy M Romesser PB. Association of Ablative Radiation Therapy With Survival Among Patients With Inoperable Pancreatic Cancer. JAMA Oncol (2021) 7(5):735–8. doi: 10.1001/jamaoncol.2021.0057 PMC7953335 33704353

[9] Rudra S Jiang N Rosenberg SA Olsen JR Roach MC Wan L. Using Adaptive Magnetic Resonance Image-Guided Radiation Therapy for Treatment of Inoperable Pancreatic Cancer. Cancer Med (2019) 8(5):2123–32. doi: 10.1002/cam4.2100 PMC6536981 30932367

[10] Hassanzadeh C Rudra S Bommireddy A Hawkins WG Wang-Gillam A Fields RC. Ablative Five-Fraction Stereotactic Body Radiotherapy for Inoperable Pancreatic Cancer Using Online MR-Guided Adaptation. Adv Radiat Oncol (2020) 6(1):100506. doi: 10.1016/j.adro.2020.06.010 33665480 PMC7897757

[11] Chuong MD Bryant J Mittauer KE Hall M Kotecha R Alvarez D. Ablative 5-Fraction Stereotactic Magnetic Resonance-Guided Radiation Therapy With On-Table Adaptive Replanning and Elective Nodal Irradiation for Inoperable Pancreas Cancer. Pract Radiat Oncol (2021) 11(2):134–47. doi: 10.1016/j.prro.2020.09.005 32947042

[12] Henke L Kashani R Robinson C Curcuru A DeWees T Bradley J. Phase I Trial of Stereotactic MR-Guided Online Adaptive Radiation Therapy (SMART) for the Treatment of Oligometastatic or Unresectable Primary Malignancies of the Abdomen. Radiother Oncol (2018) 126(3):519–26. doi: 10.1016/j.radonc.2017.11.032 29277446

[13] Rodriguez LL Kotecha R Tom MC Chuong MD Contreras JA Romaguera T. CT-Guided Versus MR-Guided Radiotherapy: Impact on Gastrointestinal Sparing in Adrenal Stereotactic Body Radiotherapy. Radiother Oncol (2021) 166:101–9. doi: 10.1016/j.radonc.2021.11.024 34843842

[14] Chuong MD Herrera R Chundru S Gutierrez A Romaguera T Alvarez D. Cumulative Target Volume Dose and Locoregional Failure in Pancreatic Cancer Patients With Treated With Ablative Stereotactic MR-Guided Adaptive Radiation Therapy (SMART). Int J Radiat Oncol Bio Phys (2021) 111(3):S141. doi: 10.1016/j.ijrobp.2021.07.318

[15] Tempero MA Malafa MP Al-Hawary M Behrman SW Benson AB Cardin DB. Pancreatic Adenocarcinoma, Version 2.2021, NCCN Clinical Practice Guidelines in Oncology. J Natl Compr Canc Netw (2021) 19(4):439–57. doi: 10.6004/jnccn.2021.0017 33845462

[16] Teriaca MA Loi M Suker M Eskens F van Eijck CHJ Nuyttens JJ. A Phase II Study of Stereotactic Radiotherapy After FOLFIRINOX for Locally Advanced Pancreatic Cancer (LAPC-1 Trial): Long-Term Outcome. Radiother Oncol (2021) 155:232–6. doi: 10.1016/j.radonc.2020.11.006 33217500

[17] Mellon EA Hoffe SE Springett GM Frakes JM Strom TJ Hodul PJ. Long-Term Outcomes of Induction Chemotherapy and Neoadjuvant Stereotactic Body Radiotherapy for Borderline Resectable and Locally Advanced Pancreatic Adenocarcinoma. Acta Oncol (2015) 54(7):979–85. doi: 10.3109/0284186X.2015.1004367 25734581

[18] Iacobuzio-Donahue CA Fu B Yachida S Luo M Abe H Henderson CM. DPC4 Gene Status of the Primary Carcinoma Correlates With Patterns of Failure in Patients With Pancreatic Cancer. J Clin Oncol (2009) 27(11):1806–13. doi: 10.1200/JCO.2008.17.7188 PMC2668706 19273710

[19] Mahadevan A Moningi S Grimm J Li XA Forster KM Palta M. Maximizing Tumor Control and Limiting Complications With Stereotactic Body Radiation Therapy for Pancreatic Cancer. Int J Radiat Oncol Biol Phys (2021) 110(1):206–16. doi: 10.1016/j.ijrobp.2020.11.017 33358561

[20] Hammel P Huguet F van Laethem JL Goldstein D Glimelius B Artru P. Effect of Chemoradiotherapy vs Chemotherapy on Survival in Patients With Locally Advanced Pancreatic Cancer Controlled After 4 Months of Gemcitabine With or Without Erlotinib: The LAP07 Randomized Clinical Trial. JAMA (2016) 315(17):1844–53. doi: 10.1001/jama.2016.4324 27139057

[21] Palta M Godfrey D Goodman KA Hoffe S Dawson LA Dessert D. Radiation Therapy for Pancreatic Cancer: Executive Summary of an ASTRO Clinical Practice Guideline. Pract Radiat Oncol (2019) 9(5):322–32. doi: 10.1016/j.prro.2019.06.016 31474330

[22] Miller JA Toesca DAS Baclay JRM Vitzthum LK Dubrowski P Pollom EL. Pancreatic Stereotactic Body Radiation Therapy With or Without Hypofractionated Elective Nodal Irradiation. Int J Radiat Oncol Biol Phys (2021) 112(1):131–42. doi: 10.1016/j.ijrobp.2021.07.1698 34348171

[23] Chuong MD Kharofa J Sanford NN. Elective Target Coverage for Pancreatic Cancer: When Less Does Not Clearly Achieve More. Int J Radiat Oncol Biol Phys (2022) 112(1):143–5. doi: 10.1016/j.ijrobp.2021.08.024 34919872

[24] Hall WA Paulson ES van der Heide UA Fuller CD Raaymakers BW Lagendijk JJW. The Transformation of Radiation Oncology Using Real-Time Magnetic Resonance Guidance: A Review. Eur J Canc (2019) 122:42–52. doi: 10.1016/j.ejca.2019.07.021 PMC8447225 31614288

[25] Magallon-Baro A Milder MTW Granton PV Nuyttens JJ Hoogeman MS. Comparison of Daily Online Plan Adaptation Strategies for a Cohort of Pancreatic Cancer Patients Treated With SBRT. Int J Radiat Oncol Biol Phys (2021) 111(1):208–19. doi: 10.1016/j.ijrobp.2021.03.050 33811976

[26] Tominaga H Matsuzaki J Oikawa C Toyoshima K Manabe H Ozawa E. Challenges for Better Diagnosis and Management of Pancreatic and Biliary Tract Cancers Focusing on Blood Biomarkers: A Systematic Review. Cancers (Basel) (2021) 13(16):1–12. doi: 10.3390/cancers13164220 PMC8394661 34439378

[27] Ferrone CR Marchegiani G Hong TS Ryan DP Deshpande V McDonnell EI. Radiological and Surgical Implications of Neoadjuvant Treatment With FOLFIRINOX for Locally Advanced and Borderline Resectable Pancreatic Cancer. Ann Surg (2015) 261(1):12–7. doi: 10.1097/SLA.0000000000000867 PMC4349683 25599322

[28] Jolissaint JS Reyngold M Bassmann J Seier KP Gonen M Varghese AM. Local Control and Survival After Induction Chemotherapy and Ablative Radiation Versus Resection for Pancreatic Ductal Adenocarcinoma With Vascular Involvement. Ann Surg (2021) 274(6):894–901. doi: 10.1097/SLA.0000000000005080 34269717 PMC8599622

